# Factors associated with stroke among patients with hypertension in Eastern Ethiopia: a case-control study

**DOI:** 10.1186/s12883-026-04776-x

**Published:** 2026-03-03

**Authors:** Fentahun Meseret, Ayichew Alemu, Tilahun Teshager, Henok Legesse, Melaku Getachew, Yalew Mossie, Fenta Wondimneh

**Affiliations:** 1 College of Health and Medical Science, School of Nursing, Haramaya University, P.O. Box 235, Harar, Ethiopia; 2 Department of Emergency and Critical Care Nursing, College of Health and Medical Sciences, Haramaya University, Harar, Ethiopia; 3 College of Health and Medical Science, School of Medicine, Haramaya University, P.O. Box 235, Harar, Ethiopia

**Keywords:** Stroke, Hypertension, Associated factors, Eastern Ethiopia

## Abstract

**Background:**

Stroke is becoming more commonplace worldwide at an incredible pace. Evidence on stroke among hypertensive patients in low-resource settings is limited, and identified risk vary across populations. Therefore, the purpose of this study was to identify factors associated with stroke among hypertensive patients in eastern Ethiopia.

**Methods:**

A hospital based case-control study was conducted from October 1, 2022, to November 30, 2023. Adult hypertensive patients with strokes were included as cases, while hypertensive patients without strokes served as controls. Cases and controls were identified through medical record review. In this study, 107 cases and 214 controls were selected by a systematic random sample technique. Data were collected through interviews, physical measurements, and record review. Stata version 17 was used to analyze the data after it was entered into Epi Data version 4.6. Variables with a p-value < 0.25 in bivariate analysis were included in the multivariable logistic regression model. Statistical significance was declared at *p* < 0.05 using adjusted odds ratios (AORs) with 95% confidence intervals (CIs).

**Results:**

The mean age of cases and controls was 62.9 years (SD ± 13.3) and 49.8 years (SD ± 14.7) respectively. Increasing age (AOR = 1.05, 95%CI: 1.02–1.07), low oxygen saturation (AOR = 0.87, 95%CI: 0.76–0.98), diabetes mellitus (AOR = 2.77, 95%CI: 1.37–5.60), current alcohol consumption (AOR = 3.48, 95%CI: 1.48–8.15) and poor knowledge of hypertension (AOR = 0.41, 95%CI: 0.21–0.82) were significantly associated with stroke.

**Conclusion:**

Stroke among hypertensive patients was associated with older age, reduced oxygen saturation, diabetes mellitus, alcohol consumption, and poor knowledge of hypertension. Strengthening health education on modifiable risk factors and integrating targeted lifestyle counseling into routine follow-up care are essential for improving primary stroke prevention.

**Supplementary Information:**

The online version contains supplementary material available at 10.1186/s12883-026-04776-x.

## Background

Stroke is a chronic non-communicable disease (NCD) characterized by sudden neurological impairment due to reduced cerebral perfusion [1]. It is a major global health challenge and ranks as the fourth leading cause of mortality and disability worldwide [1, 2]. Stroke can result in various social and cognitive consequences, including communication difficulty, memory loss, depression, walking difficulty, and paralysis [3]. It originates from blockage or rupture of cerebral blood vessels, leading to ischemic or hemorrhagic events [4]. Globally, there are approximately 15 million stroke cases annually, causing 5.8 million deaths, with survivors often experiencing significant motor and non-motor impairments that affect daily functioning [2–4].

Hypertension (HTN) is the leading modifiable factors for stroke, contributing about 70% of ischemic strokes worldwide [5, 6]. Despite differences in prevalence between high and low income countries, nearly 40% of the global population is affected by HTN [7]. Poor awareness, inadequate diagnosis, and suboptimal management of HTN increase the risk of stroke [8]. Studies in Ethiopia report that 58–72% of hypertensive patients are unaware of stroke risk factors or warning signs [9]. Other global risk factors include age, sex, smoking, low physical activity, obesity, alcohol use, medication non-adherence, uncontrolled blood pressure, diabetes, and high cholesterol [10].

The burden of stroke is disproportionately high in low-income countries, accounting for 75% of stroke and 81% of stroke-related disability-adjusted life years [11]. It also imposes substantial economic costs, with losses exceeding $7 trillion globally between 2011 and 2025, particularly in LMICs [12]. In Ethiopia, stroke is a major public health problem, accounting for 18% of all deaths, with ischemic and hemorrhagic subtypes contributing significantly to hospital admissions and mortality [13].

Effective stroke prevention strategies, including lifestyle modifications, and early recognition of warning signs could prevent up to 80% of strokes [14, 15]. National and international initiatives, such as the American Heart Association’s recommendations and Ethiopia’s health sector development plan, emphasize controlling blood pressure, promoting healthy lifestyles, and improving public awareness to reduce stroke-related morbidity and mortality [12, 16]. Despite the rising burden of stroke among hypertensive patients in Ethiopia, data on associated risk factors remain limited. Therefore, this study aimed to assess the factors associated with stroke among hypertensive patients in four selected public hospitals in eastern Ethiopia.

## Materials and methods

### Study setting and period

This study was conducted in adult emergency departments (EDs) and follow-up clinics of four public hospitals in eastern Ethiopia: Hiwot Fana Comprehensive Specialized University Hospital (Harari Regional State), Dilchora Referral Hospital (Dire Dawa City Administration), Sheik Hassen Yabare Specialized University Hospital (Somali Regional State) and Haramaya General Hospital (East Hararghe Zone, Oromia Regional State).

The study period extended from October 1, 2022, to November 30, 2023.

### Study design

Hospital based unmatched case-control study was conducted to identify determinants of stroke. Cases were adult patients who presented to the ED or follow up clinic of the selected public hospitals with a diagnosed of stroke made by a neurologist (consultant internist) and confirmed by brain imaging, including compute tomography (CT-scan) or magnetic resonant image (MRI). Stroke cases were classified as ischemic or hemorrhagic based on clinical assessment and neuroimaging findings.

Controls were sampled adult hypertensive patients without clinical evidence of stroke who attended the same public hospitals during the study period.

### Populations

#### Source populations

All adult hypertensive patients attending the EDs and follow-up clinics of public hospitals in eastern Ethiopia.

### Study populations

All adult hypertensive patients who visited the EDs and follow-up clinics of the selected hospitals during the study period.

### Eligibility criteria

Adult hypertensive patients with stroke were included as cases, while adult hypertensive patients without stroke were included as controls. Patients younger than 18 years, those who were mentally incompetent or critically ill during data collection, pregnant women, cases with fewer than three hypertension follow-up visits before the first stroke event, and controls with fewer than three follow-up visits were excluded.

### Sample size determination

The sample size was calculated using Epi Info version 7.0 based on factors significantly associated with stroke in previous studies. The factor yielding the largest sample size was selected. Assumptions included a 95% confidence level, 80% power, a case-to-control ratio of 1:2, and a 5% margin of error. Using an exposure proportion of 94.1% among cases and 83% among controls, with an adjusted odds ratio of 3.25 from a prior study [16], the calculated sample size was 305 (102 cases and 203 controls). After adding 5% for non-response, the final sample size was 321 (107 cases and 214 controls).

### Sampling technique and procedures

**Fig. 1:**
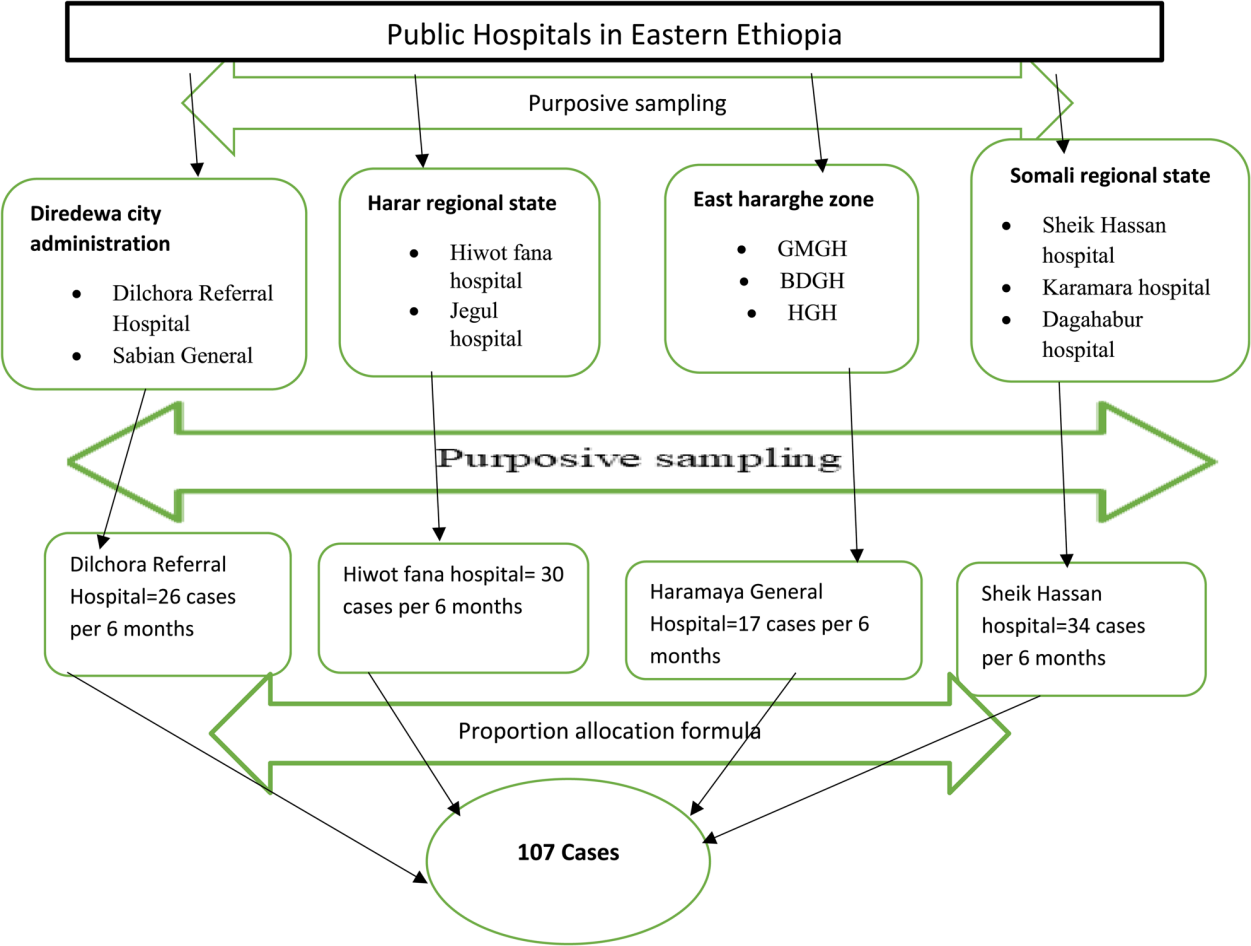
*Sampling techniques and proportional allocation of sample* to identify factors associated with stroke among hypertensive patients in eastern Ethiopia, 2024

The study was conducted at four selected public hospitals. Based on hospital HMIS and ED registry data, 162 stroke cases were reported in the preceding six months. Cases and controls were allocated proportionally to each hospital. Eligible participants were recruited using a systematic random sampling technique. For each identified case, two consecutive eligible controls were selected until the required sample size was achieved. (Fig. 1).

### Data collection tools and procedure

Data were collected using a structured and pretested questionnaire adapted from previous studies with some modification [12, 16, 17]. The questionnaire consisted of four sections: socio-demographic characteristics, vital signs, comorbidities, and behavioral and clinical factors, including medication adherence and knowledge of hypertension. The questionnaire was translated into Amharic, Afan Oromo, and Afsomali, then back-translated into English to ensure consistency. Data were collected through face-to-face interviews, physical measurements, and medical record reviews.

Data collection was conducted over 12 months by 16 trained BSc nurses (four per hospital), supervised by four emergency and critical care nurses. One trained data clerk supported data management. Patient diagnoses, vital signs, and comorbidities were extracted from medical records.

### Dependent variables

Stroke.

### Independent variables

Socio-demographic factors (age, sex, marital status, occupation, residence, and educational status), behavioral risk factors (physical exercise, smoking, alcohol consumption, frequency of follow-up, dietary salt intake, fatty food use, loss to follow-up, medication adherence), and clinical factors (duration of hypertension, diabetes mellitus, blood pressure control, BMI, fasting blood glucose, cholesterol level, and comorbidities).

### Operational definitions and measurements

Weight was measured in light closing and without shoes by calibrated digital weighing scale. Stadiometer in centimeter in erect position at a precision of 0.1 cm without shoes was used to measure height. Mercury sphygmomanometer was used to measure blood pressure; an average of two measurements 5–10 min apart was recorded as final data.

*Physically active*: if patients make regular physical activities 30 min and above, 5 days and above per week, physically inactive: if patient is made physical exercise less than 30 min or less than 5 days per week.

 Medication adherence to anti-hypertensive treatment was assessed using the 8-item Morisky Medication Adherence Scale (MMAS), a validated and widely used tool. Each item was scored as Yes = 0 and No = l, with a total score ranging from 0 to 8. Participants scoring 7–8 were considered adherent, while those scoring < = 6 were considered non-adherent [17]. Where available, self-reported adherence was cross-checked with medical records and prescription refill history.

*Alcohol consumption*: was assessed using self-report, and participants were classified as alcohol drinkers if they consumed ≥ 10.5 units of alcohol per week, based on standard definitions [18, 19]. To reduce recall bias, participants were assisted with local drink conversion charts during data collection.

Physical measurements and clinical factors including: Fasting blood glucose (FBG), cholesterol level, blood pressure control, body mass index (BMI) and comorbidities have been also collected. Normal FBG = 126 mg/dl [18, 19]. Cholesterol level: normal if less than 200 and high cholesterol level 200 and above, BMI: underweight (less than 18.5), normal (18.5–24.9), overweight (25–29.9) and obese (30 and above). Systolic blood pressure: controlled (= 140), diastolic blood pressure: controlled (= 90) [19].

### Data quality control

Data quality control was maintained by different data quality control mechanisms. The English language questionnaire was first translated into the local languages (Amharic, Afan Oromo and Afsomali) by Language experts and was then back translated to English to maintain consistency. The questionnaire have checked for its coherence & completeness. Pretesting of the questionnaire was done on 5% of the sample size at Jegol General Hospital, to enhance reliability of the instrument. Training have given for data collectors, the data clerk and supervisor prior to the actual data collection is started for two days. Principal investigator and supervisors made spot-checking and reviewing the completed questionnaires to ensure completeness and consistency of the information collected.

Missing data were assessed for each variable before analysis. Variables with missing values of less than 5% were handled using complete case analysis, excluding only cases with missing responses for that variable. Outliers were identified using boxplots and extreme value checks, and any inconsistencies were verified against source documents to ensure accuracy. These procedures ensured high data quality and reliability of the results.

### Data processing and analysis

Data were entered into EpiData version 4.6 and analyzed using Stata version 17. Descriptive statistics were computed. Variables with p-values < 0.25 in bivariate logistic regression were entered into the multivariable model. Model fitness was assessed using the Hosmer–Lemeshow test, and multicollinearity was checked using variance inflation factors. Adjusted odds ratios with 95% confidence intervals were used to identify independent predictors of stroke, with statistical significance set at *p* < 0.05.

## Results

### Socio-demographic characteristics of the study participants

All 321 selected study participants (107 cases and 214 controls) have participated in the study and the response rate was 100%. The mean age of cases was 62.9 years (SD ± 13.3), while that of controls was 49.8 years (SD ± 14.7).

The majority of participants were married, accounting for 65.4% of cases and 64.0% of controls. Seventy-nine (73.8%) cases and 165(77.1%) controls were employed. Monthly income greater than 5000 Ethiopian birr was reported by 73 (68.2%) cases and 119 (55.6%) controls (Table 1).

**Table 1:** Socio demographic characteristics of the study participants to identify factors associated with stroke among hypertensive patients in Eastern Ethiopia, 2024

**Variables**	**Categories**	**Cases**		**Controls**	
		**Frequency**	**Percentage**	**Frequency**	**Percentage**
Age in years	18–45	12	11.2	93	43.5
	46–65	55	51.4	88	41.1
	> 65	40	3.7	33	15.4
Sex	Male	72	67.3	132	61.7
	Female	35	32.7	82	38.3
Religion	Orthodox	34	31.8	63	29.4
	Muslim	61	57	134	62.6
	Protestant	6	5.6	14	6.5
	Catholic	4	3.7	2	0.9
	Other	2	1.9	1	0.5
Marital status	Single	14	13.1	48	22.4
	Married	70	65.4	137	64
	Divorce	12	11.2	13	6.1
	Widowed	11	10.3	16	7.5
Educational status	No formal education	17	15.9	45	21
	Primary school	9	8.4	28	13.1
	Secondary school	19	17.8	34	15.9
	College and above	62	57.9	107	50
Occupation	Unemployed	28	26.2	49	22.9
	Employed	79	73.8	165	77.1
Residency	Urban	41	38.3	95	44.4
	Rural	66	61.7	119	55.6
Income	< 1000	8	7.5	32	15
	1000–5000	26	24.3	63	29.4
	> 5000	73	68.2	119	55.6

### Behavioral factors of the study participants

Among the participants, 95 (88.8%) cases and 143 (66.8%) controls reported not consuming fatty diets. Twenty-two (20.6%) cases and 63 (29.4%) controls did not reduce salt intake in their diet. Based on self-report, 84.1% of cases and 73.4% of controls reported taking their medications correctly.

Regular physical exercise was reported by 85 (79.4%) cases and 151 (70.6%) controls. A history of loss to follow-up was reported by 53 (49.5%) cases and 62 (29.0%) controls (Table 2).

**Table 2:** Behavioral related factors of the study participants to identify factors associated with stroke among hypertensive patients in Eastern Ethiopia, 2024

Variables	Categories	Cases		Controls	
		Frequency	percentage	Frequency	Percentage
Have you ever smoke cigarettes	Yes	65	60.7	88	41.1
	NO	42	39.3	126	58.9
Regular physical exercise	Yes	85	79.4	151	70.6
	NO	22	20.6	63	29.4
Knowledge	Good	37	34.6	133	62.1
	Poor	70	65.4	81	37.9
Adherence	Good	58	54.2	32	15
	Medium	20	18.7	55	25.7
	Poor	29	27.1	127	59.3
Took pills correctly	Yes	90	84.1	157	73.4
	No	17	15.9	57	26.6
Current alcohol	Yes	50	46.7	31	14.5
	NO	57	53.3	183	85.5
Salt in diet	Yes	85	79.4	146	68.2
	NO	22	20.6	68	31.8
Avoid fatty foods	Yes	95	88.8	143	66.8
	NO	12	11.2	71	33.2
Chat chewing	Yes	73	68.2	144	67.3
	NO	34	31.8	70	32.7
Regular follow up	Yes	45	42.1	67	31.3
	NO	53	49.5	62	29

### Clinical assessment and characteristics of the study participants

Vital signs, including pulse rate (PR), blood pressure (BP), and oxygen saturation (SpO₂), were assessed for all participants. Elevated blood pressure was observed in 34 (31.8%) cases and 117 (54.7%) controls. The mean PR and SpO₂ were 109.7 ± 25.3 beats/min and 93.8 ± 2.7% among cases, and 97.4 ± 15.8 beats/min and 94.3 ± 2.9% among controls, respectively.

Regarding clinical history, 65.4% of cases and 46.3% of controls reported a family history of hypertension, while 4.7% of cases and 0.9% of controls reported a family history of stroke. The mean duration of hypertension was 4.5 ± 4.2 years among cases and 3.7 ± 4.3 years among controls.

The mean total cholesterol level was 188 ± 24 mg/dL among cases and 172 ± 17 mg/dL among controls. The mean systolic blood pressure was 182.4 ± 22.2 mmHg in cases and 177.8 ± 27.0 mmHg in controls, while the mean diastolic blood pressure was 113.0 ± 20.2 mmHg and 109.8 ± 15.7 mmHg, respectively (Table 3).

**Table 3:** Clinical profile of the study participants to identify factors associated with stroke among hypertensive patients in Eastern Ethiopia, 2024

**Variables**	**Categories**	**Cases**		**Controls**	
		**Frequency**	**Percentage**	**Frequency**	**Percentage**
Duration since diagnosis	< 4 year	73	68.2	157	73.4
	>=4 year	34	31.8	57	26.6
SPO2	Normal	97	90.7	172	80.4
	Desaturated	10	9.3	42	19.6
Cholesterol level	Normal	86	80.4	181	84.6
	High level	21	19.6	33	15.4
Blood glucose level	Normal	46	43	175	81.8
	High level	61	57	39	18.2
Over weight	Yes	39	36.4	138	64.5
	NO	68	63.6	76	35.5
Increased BP	Yes	34	31.8	117	54.7
	NO	73	68.2	97	45.3
SBP	Controlled	2	1.9	5	2.3
	Uncontrolled	105	98.1	209	97.7
DBP	Controlled	10	9.3	15	7
	Uncontrolled	97	90.7	199	93
Family history of HTN	Yes	70	65.4	99	46.3
	NO	37	34.6	115	53.7
Family history of stroke	Yes	5	4.7	2	0.9
	NO	102	95.3	212	99.1
Comorbidities	Yes	105	98.1	96	44.9
	NO	2	1.9	116	54.2
DM	Yes	61	57	39	18.2
	NO	46	43	175	81.8

Where, *SBP* systolic blood pressure, *DBP* diastolic blood pressure, *SPO2* oxygen saturation, *DM* diabetus militus, *HTN* hypertention

### Stroke classification and methods of approach used for diagnosis

Based on medical record review, strokes were classified as ischemic or hemorrhagic. Among the 107 cases, 63 (58.9%) had ischemic stroke, of whom 26 had cardioembolic stroke, while 44 (41.1%) had hemorrhagic stroke. Computed tomography (CT) scan was the most commonly used diagnostic modality, followed by clinical diagnosis and magnetic resonance imaging (MRI).

### Bivariable and multivariable logistic regression for factors associated with stroke among hypertensive patients

In the bivariable logistic regression analysis, age, educational status, marital status, income, systolic and diastolic blood pressure, pulse rate, oxygen saturation, diabetes mellitus, family history of hypertension, current alcohol consumption, excessive salt intake, fatty diet consumption, medication adherence, overweight status, regular physical exercise, and knowledge of hypertension were significantly associated with stroke.

However, in the multivariable logistic regression analysis, only age (AOR = 1.05, 95% CI: 1.02–1.07), oxygen saturation (AOR = 0.87, 95% CI: 0.78–0.98), diabetes mellitus (AOR = 2.77, 95% CI: 1.37–5.60), current alcohol consumption (AOR = 3.48, 95% CI: 1.48–8.15), and good knowledge of hypertension (AOR = 0.41, 95% CI: 0.21–0.82) remained independently associated with stroke among hypertensive patients (Table 4).

**Table 4:** Bivariable and multivariable logistic regression to identify factors associated with stroke among hypertensive patients in Eastern Ethiopia, 2024

Variables	Category	Cases (%)	Controls (%)	COR(95%CI)	AOR(95%CI)	*p*-value
Age	>65 years	40(3.7)	33(15.4)	1.07(1.05, 1.09)	1.05(1.02,1.07)	0.001*
SBP	Uncontrolled	105(98.1)	209(97.7)	1.01(0.99, 1.02)	0.99(0.97,1.02)	0.68
DBP	Uncontrolled	97(90.7)	199(93)	1.01(0.99, 1.02)	1.00(0.97,1.03)	0.87
SPO2	Normal	97(90.7)	172(80.4)	0.93(0.86, 1.01)	0.87(0.78,0.98)	0.019*
Education	Primary school^®^	9(8.4)	28(13.1)			
	Secondary school	19(17.8)	34(15.9)	1.74(0.68, 4.44)	2.41(0.80,7.25)	0.116
	College and above	62(57.9)	107(50.0)	1.80(0.79, 4.06)	1.02(0.38,2.76)	0.962
	No formal education	17(15.9)	45(21.0)	1.18(0.46, 2.99)	1.26(0.42,3.79)	0.682
Marital status	Single^®^	14(13.1)	48(22.4)			
	Married	70(65.4)	137(64.0)	1.75(0.90, 3.39)	0.89(0.37,2.18)	0.814
	Divorced	12(11.2)	13(6.1)	3.16(1.18, 8.47)	0.56(0.13,2.36)	0.427
	Widowed	11(10.3)	16(7.5)	2.36(0.89, 6.23)	0.74(0.21,2.68)	0.653
DM	Yes	61(57.0)	39(18.2)	5.95(3.55, 9.98)	2.77(1.37,5.60)	0.005*
	NO^®^	46(43.0)	175(81.8)			
Family history of HTN	Yes	37(34.6)	115(53.7)	0.46(0.28, 0.74)	0.67(0.35,1.26)	0.214
	NO^®^	70(65.4)	99(46.3)			
Smoking	Yes	65(60.7)	88(41.1)	2.22(1.38, 3.56)	0.84(0.43,1.67)	0.627
	NO^®^	42(39.3)	126(58.9)			
Use of salty food	Yes	85(79.4)	146(68.2)	1.79(1.04, 3.12)	1.48(0.74,2.97)	0.271
	NO^®^	22(20.6)	68(31.8)			
Regular exercise	Yes	85(79.4)	151(70.6)	1.61(0.93, 2.80)	1.49(0.71,3.11)	0.294
	NO^®^	22(20.6)	63(29.4)			
Current alcohol drinker	Yes	50(46.7)	31(14.5)	5.18(3.02, 8.87)	3.48(1.48,8.15)	0.004*
	NO^®^	57(53.3)	183(85.5)			
Knowledge of HTN	Good	37(34.6)	133(62.1)	0.32(0.19, 0.52)	0.41(0.21,0.82)	0.012*
	Poor^®^	70(65.4)	81(37.9)			

*COR* crude odds ratio, *AOR* adjusted odds ratio, *SBP* systolic blood pressure, *DBP* diastolic blood pressure, *SPO2* oxygen saturation, *DM* diabetus militus, *HTN* hypertention

*indicates statistically significant variable with multivariable logistic regression, and ^®^indicatesreference category

## Discussion

This hospital-based case–control study identified factors associated with stroke among hypertensive patients attending four public hospitals in eastern Ethiopia. Age, oxygen saturation, diabetes mellitus, current alcohol consumption, and knowledge of hypertension were independently associated with stroke.

In this study, as patients age increases by one year, the probability of developing stroke also increases by 1.05; which means that, aging is the strongest non modifiable risk factor for stroke occurrence among hypertensive patients. The finding is supported by the study conducted in Cameron [20], Japan [21], China [22], Bangladesh [23], Croatian urban area [24], Italy [25], university of Texas [26]. The increased risk with advancing age may be explained by age-related degenerative changes and immune system alterations that promote atherosclerosis, vascular stiffness, and endothelial dysfunction, thereby increasing susceptibility to ischemic and hemorrhagic stroke [27, 28]. In addition, longer duration of hypertension with increasing age may further elevate stroke risk [29].

Oxygen saturation was inversely associated with stroke, such that a one-percent increase in SpO₂ reduced the odds of stroke by 13%. The finding is in line with the study carried out in south Africa where, hypertensive patients demonstrated significantly greater requirements for oxygen to decrease the occurrence of such devastating complication even including death [30]. This is due to the fact that, if the blood has low level of oxygen, it cannot deliver enough oxygen and other important nutrients to the target organs and tissues that need to keep its normal physiology; this in turn, can damage the target organs like heart and brain specially, if it persists over a long period of time [31]. This highlights the importance of routine monitoring of oxygen saturation in hypertensive patients, especially those with comorbid conditions. Low-cost interventions such as pulmonary assessment, early detection of respiratory compromise, and tailored physical activity programs may help improve oxygenation in resource-limited settings like Ethiopia [32].

Current alcohol consumption was strongly associated with stroke. Hypertensive patients who consumed alcohol were more than three times more likely to develop stroke compared to non-drinkers. This finding is similar to the previous study reported in Ethiopia [16], Nigeria [33], Bangladesh [23], Croatian urban area [24], China [5], India [34], and in 32 countries (INTERSTROKE) [35]. This is due to the causal association between alcohol consumption and increment of blood pressure/hypertension [36, 37], and consistent with recommendations to avoid or limit alcohol intake [38, 39]. In addition to hypertension, alcohol consumption can also increases the occurrence of other chronic diseases such as DM, cirrhosis of liver, lung disease and gastritis which further increases the risk [40].

Diabetes mellitus was another significant predictor of stroke. Hypertensive patients with diabetes had nearly three times higher odds of stroke compared to those without diabetes. This finding is consistent with a study conducted in Japan [21], Bangladesh [23], Saudi Arabia [41], India [42], Republic of Korea [43], Italy [44], and 22 countries (the INTERSTROKE study) [35]. Hyperglycemia and insulin resistance accelerate atherosclerosis and promote prothrombotic states, increasing the risk of both ischemic and hemorrhagic stroke [45]. These findings emphasize the need for integrated management of hypertension and diabetes, including regular screening for vascular complications, particularly in low- and middle-income countries such as Ethiopia [46].

Good knowledge of hypertension was found to be protective against stroke. Participants with adequate knowledge were 59% less likely to develop stroke compared to those with poor knowledge. This finding is in lines with a study done in Ethiopia [47], Ghana [48], Indonesia [49], Nepal [50], Austria [51], Ireland [52], and china [53]. Improved knowledge likely enhances medication adherence, lifestyle modification, and health-seeking behavior, all of which contribute to better blood pressure control and stroke prevention [54]. Strengthening patient education through structured health education programs during routine follow-up visits could substantially reduce the burden of stroke among hypertensive patients [55, 56].

The findings of this study have important implications for stroke prevention among hypertensive patients in Ethiopia. Given the strong association of modifiable factors such as alcohol consumption, diabetes mellitus, oxygen saturation, and knowledge of hypertension with stroke, targeted preventive strategies should be integrated into routine hypertension care. Health care providers, particularly nurses and physicians working in outpatient and emergency settings, should routinely assess lifestyle behaviors, oxygen saturation status, and comorbid conditions during follow-up visits.

Strengthening patient education on hypertension self-care, medication adherence, and lifestyle modification may significantly reduce stroke risk, especially in resource-limited settings. Incorporating structured health education programs and task-shifting strategies through health extension workers could enhance patient awareness and long-term behavioral change. In addition, integrated management of hypertension and diabetes, including early screening and continuous follow-up, should be prioritized to reduce vascular complications. These findings support the need for strengthening existing non-communicable disease prevention strategies within Ethiopia’s health system to reduce stroke-related morbidity and mortality.

## Conclusion

Among hypertensive patients, Age, oxygen saturation, diabetes mellitus, current alcohol consumption, and knowledge of hypertension were significantly associated with stroke. Strengthening routine health education on modifiable risk factors, promoting lifestyle modification, and improving medication adherence during each follow-up visit are essential for primary stroke prevention. Furthermore, integrated management of hypertension diabetes within existing health services is crucial to reduce the burden of stroke in Ethiopia.

### Strength of the study

This study has several strengths. First, stroke diagnosis was based on clinical assessment and confirmation by neuroimaging (CT scan or MRI), which enhances diagnostic accuracy. Second, the inclusion of multiple public hospitals increases the representativeness of the study population within the region. Third, the study focuses on hypertensive patients in a low-income setting, where evidence on stroke determinants remains limited.

The findings of this study are consistent with reports from other populations, which identify hypertension, poor blood pressure control, and lifestyle-related factors as major contributors to stroke risk. However, differences in the magnitude and distribution of risk factors compared with studies from high-income countries may reflect variations in healthcare access, awareness, socioeconomic status, and preventive practices. These similarities and differences underscore the importance of context-specific prevention strategies.

### Limitations of the study

This study has several limitations that should be considered when interpreting the findings. First, controls were recruited from hypertensive patients attending public hospitals rather than from the general population, which may have introduced selection bias and limited the generalizability of the results. Future studies should consider recruiting controls from the general population to better reflect baseline exposure distributions.

Second, due to the unmatched case–control design, residual confounding from unmeasured or incompletely measured variables may remain despite adjustment in multivariable analysis. Important potential confounders such as genetic predisposition, psychosocial stress, dietary patterns, and nutritional status were not assessed in this study and may have influenced the observed associations.

Third, the hospital-based nature of the study may limit the applicability of the findings to other populations, particularly community-based settings. Fourth, the relatively limited sample size may have reduced the statistical power to detect weaker associations. Fifth, the retrospective case–control design precludes assessment of temporal relationships and causal inferences; therefore, prospective longitudinal studies are warranted to confirm these findings. Sixth, although validated tools were used to assess behavioral variables, alcohol consumption and some lifestyle behaviors were self-reported and may be subject to recall and social desirability bias.

Finally, the severity of stroke at presentation (e.g., NIH Stroke Scale or Glasgow Coma Scale scores) was not systematically captured and analyzed, which limited the ability to assess the relationship between identified risk factors and stroke severity. Future studies should incorporate standardized stroke severity measures to provide more comprehensive clinical insights.

## Supplementary Information

Supplementary Material 1.

## Data Availability

The data that support the findings of this study are available from the corresponding author upon reasonable request.

## Abbreviations

AOR: Adjusted Odds Ratio
COR: Crude Odds Ratio
CVA: Cerebrovascular Accident
DBP: Diastolic Blood Pressure
DM: Diabetes Miletus
ED: Emergency Department
HFCSUH: Hiwot Fana Comprehensive Specialized University Hospital
HTN: Hypertension
LMIC: Low and Middle Income Countries
NCD: Non Communicable Disease
PR: Pulse Rate
SPO2: Partial Oxygen Saturation
SBP: Systolic Blood Pressure
WHO: World Health Organization
