# Supplementary material for: Factors associated with stroke among patients with hypertension in Eastern Ethiopia: a case-control study
Source: BMC Neurol. 2026 Mar 3;26:224. doi: 10.1186/s12883-026-04776-x (PMC13063997; doi:10.1186/s12883-026-04776-x)
Supplement: Supplementary file 1 — Supplementary Material 1. [file 12883_2026_4776_MOESM1_ESM.docx]

**English Version of Questionnaires**

**Part one: Sociodemographic status**

| Ser. No | Items | Responses |
| --- | --- | --- |
| 101 | What is your age in years? | _____________ |
| 102 | What is your sex? | 1. Male 2. Female |
| 103 | What is your religion | 1. Orthodox 2. Muslim  3. Protestant 4. Catholic  5. Other |
| 104 | Where is your residency? | 1. Rural 2. Urban |
| 105 | What is your educational status? | 1. Primary school 2. Secondary school  3. College diploma 4. University degree  5. None educated 6. Others |
| 106 | What is your employment status? | 1. Unemployed 2. Self- Employed  3. Governmental Employee 4. Private Sector |
| 107 | What is your current marital status? | 1. Single 2. Married  3. Divorced 4. Widowed |
| 108 | How much is your monthly income? | _________________ETB |

**Part two: patient’s vital signs**

| Ser. No | Vital signs | Readings |
| --- | --- | --- |
| 209 | Blood pressure ( BP) | ________mmHg |
| 210 | Heart rate (PR) | ________bpm |
| 211 | Oxygen saturation (spo2) without extra oxygen supplementation | ________% |

**Part three: Comorbidities**

| Ser. No | Comorbidities | 1. Yes | 2. No |
| --- | --- | --- | --- |
| 301 | Have you ever been diagnosed with any comorbidity in the past? (if yes go to next question; if no skip to part four ) |  |  |
| 302 | Which of the following comorbidities you diagnosed with? | Multiple answers are possible | |
| 303 | Diabetes mellitus |  |  |
| 304 | Pulmonary HTN |  |  |
| 305 | Coronary syndrome |  |  |
| 306 | Congestive heart failure |  |  |
| 307 | Kidney disease |  |  |
| 308 | Asthma |  |  |
| 309 | Hyperthyroidism |  |  |
| 310 | Migraine |  |  |
| 311 | Others |  |  |

**Part four: potential determinants**

**A. Morisky Medication Adherence Scale**

| Ser. No | Questions | 1. Yes | 2. No |
| --- | --- | --- | --- |
| 401 | Do you sometimes forget to take your medication? |  |  |
| 402 | Thinking over the past two weeks, were there any days when you did not take your medicine for reasons other than forgetting? |  |  |
| 403 | Have you ever cut back or stopped taking your medication without telling your doctor, because you felt worse when you took it? |  |  |
| 404 | When you travel or leave home, do you sometimes forget to bring along your medication |  |  |
| 405 | Did you take your medicine yesterday? |  |  |
| 406 | When you feel like your health concern is under control, do you sometimes stop taking your medicine? |  |  |
| 407 | Taking medication every day is a real inconvenience for some people. Do you ever feel hassled about sticking to your treatment plan? |  |  |
| 408 | How often do you have difficulty remembering to take all your medications? | Never (4)  Once in a while (3) Sometime(2)  Usually(1)  All the time(0) | |

**B. Patient’s knowledge of hypertension**

| No | Questions | 1.Yes | 2. No |
| --- | --- | --- | --- |
| 401 | Knowing normal values of BP as 120/80mmHg |  |  |
| 402 | Increase in BP > 140/90mmHg called HTN |  |  |
| 403 | HTN can progress along with the age |  |  |
| 404 | Both sexes have equal chance of developing HTN |  |  |
| 405 | HTN is a treatable condition |  |  |
| 406 | Risk of developing HTN if there is a family history of HTN |  |  |
| 407 | Aging is greater risk of HTN |  |  |
| 408 | Smoking is a risk factor for HTN |  |  |
| 409 | Eating fatty foods is a risk factor for HTN |  |  |
| 410 | Overweight is a risk factor for HTN |  |  |
| 411 | Regular physical exercise reduces HTN |  |  |
| 412 | More salt consumption increases BP |  |  |
| 413 | Medication is alone in controlling HTN |  |  |
| 414 | HTN can lead to life-threatening condition |  |  |

**C. Other personal behavior related determinants**

| Ser. No | Questions | 1.Yes | | 2. No | Remarks |
| --- | --- | --- | --- | --- | --- |
| 415 | Previous history of HTN? |  | |  |  |
| 416 | Previous history of stroke? |  | |  |  |
| 417 | If yes for q416, specify type of stroke |  | |  | a. Ischemic stroke  b. Hemorrhagic stroke |
| 418 | Duration of hypertension from diagnosis in years | --------------- | | |  |
| 419 | Have you started taking medications? |  | |  |  |
| 420 | Do you have regular follow up? |  | |  |  |
| 421 | Family history of HTN? |  | |  |  |
| 422 | Family history of stroke? |  | |  |  |
| 423 | Have you ever smoke cigarette? |  | |  |  |
| 424 | Are you currently smoking cigarette? |  | |  |  |
| 425 | For how long you smoke? |  | |  | (__________years) |
| 426 | How much pieces you smoke (are smoking) per day? |  | |  | (___________) |
| 427 | Have you ever drink alcohol? |  | |  | if yes go to the next question No 612 below, if no skip to 614) |
| 428 | Are you currently drinking alcohol? |  | |  |  |
| 429 | Did you drink alcohol in the past? |  | |  |  |
| 430 | What type of alcohol you drink? | (1.Beer 2. Win 3. Traditional drinks 4. Other(specify___________________)) | | | |
| 431 | Do you chew khat? |  | |  | If no skip To Q617 |
| 432 | If yes to Q615, how Often? |  | |  | 1. Every day  2. 1-3 days/wk.  3. Occasionally |
| 433 | Take your blood pressure pills correctly? |  | |  |  |
| 434 | Avoid fatty foods? |  | |  |  |
| 435 | Add salt to food when you’re cooking? |  | |  |  |
| 436 | Do you do regular physical exercise? |  | |  | if yes go to the next question No 618 below, if no skip to 620) |
| 437 | How often do you do exercise per week? | | (_______days) | | |
| 438 | What type of exercise you do? | (1.Walking 2.Jumping 3. Others (specify____) ) | | | |
| 439 | Obesity? |  | |  |  |
| 440 | Cholesterol level  HDL  LDL |  | |  |  |
| 441 | Blood glucose level  RBS  FBS |  | |  |  |
